# Supplementary material for: Extreme dispersal or human-transport? The enigmatic case of an extralimital freshwater occurrence of a Southern elephant seal from Indiana
Source: PeerJ. 2020 Sep 2;8:e9665. doi: 10.7717/peerj.9665 (PMC7474520; doi:10.7717/peerj.9665)
Supplement: Text S1 [file peerj-08-9665-s002.docx]

**Supplementary Text S1**

**Concerns with the radiocarbon measurements**

Although the specimen was directly dated (TX-1651; Valastro et al., 1988), a number of factors could bias this measurement upward or downward with an unknown magnitude. These have to do with the technology used to perform the radiometric measurement (Liquid Scintillation Counting, LSC), the material itself (apatite), and more importantly, the time at which this work was undertaken. Although the exact date at which the radiometric measurement was taken is unknown, an FMNH archival note (see Supplementary File S1) states that the date was taken “in 1972-73”. Although LSC is still used and can be a very reliable and precise method for obtaining radiocarbon dates, advances in efficiency and contamination control that have improved this technology to its modern standard mostly postdate the mid-1970’s (Polach, 1992), which is part of the reason why this radiocarbon measurement is not being used further in this study. The date list in which TX-1651 is published lists the chemical efficiency of the laboratory as 95-99% (Valastro *et al.*, 1988), meaning that 95-99% of the sample’s carbon (including ^14^C) were incorporated in the solvents from which the date would be measured. However, up to November 1973 the laboratory’s efficiency averaged 90% (Valastro, Mott Davis and Varela, 1975) meaning that approximately 10% of the material’s carbon could have been lost when prepared for the measurements. As LSC directly measures decay events from 14C atoms, loss of sample material results in decrease in the measurement precision. Other issues with early 1970’s LSC included problems with background contamination, which would have affected the accuracy of the measurement (reviewed in Polach 1992).

*Bone*. At the time of the radiocarbon measurement, bone was considered a problematic material for radiocarbon dating, particularly apatite, the mineral portion of the bone, which suffers from contamination from the environment in which the material was deposited. This contamination could bias the measurement to appear younger (Olson and Broecker, 1961) or older (Morlan *et al.*, 1990) than the age of the material. Collagen (the organic portion of the bone), was eventually found to suffer from these issues less, and became the portion of choice for dating bone, especially as better pre-treatment methods were developed (reviewed in Taylor and Bar-Yosef 2014 section 3.2). Apatite can be pre-treated to remove non-endogenous contamination, and thus yield radiocarbon measurements that reflect the age of death of the individual (Zazzo and Saliège, 2011), but the two steps of treatment that led to these more reliable measurements were developed in 1968 (Haynes, 1968) and 1980 (Haas and Banewicz, 1980). Although no pre-treatment is mentioned in the description of Tx-1651, an earlier publication of the laboratory mentioned following Haynes (1968) in dates measured around 1970 (Valastro JR and Mott Davis, 1970), and it could be assumed that they continued with the practice. It is assumed that no measurement on the organic and/or collagen portion of the bone was carried out, given the lack of reporting, despite the laboratory having previously published measurements from both apatite and collagen of bone material, and including such doubly-dated specimens in the same publication (Valastro and Davis 1970, 1975; Valastro et al. 1988). Given these concerns and the lack of knowledge of the geological context of the bone, and therefore the potential direction of any biases introduced by contamination, as well as a lack of associated collagen date or more description on the dated portion of the bone, it is difficult to consider that this radiocarbon measurement is reliable.

*Marine bone*. As the bone belonged to a marine animal, a correction should be applied to the conventional date to offset the depletion of ^14^C in the oceans relative to the atmosphere. The intensity of the reservoir effect varies, with surface waters, mixing with the atmosphere, being less depleted in 14C than the deep ocean, and is corrected by subtracting a value, ΔR, representing the age-equivalent radiocarbon depletion (Stuiver and Braziunas, 1993). ΔR varies between locations, and Correction for marine shell carbonates vary according to location, often due to the local oceanographic conditions: shell material from upwelling areas appears older due to mixing with deeper, more 14C depleted, water (Stuiver and Braziunas, 1993). However, the one of the main mechanisms of radiocarbon uptake by organisms is through diet, and in the case of mobile marine animals such as marine mammals, the level of carbon depletion is also linked to the biology and feeding ecology of the dated organism, which results in disagreements between ΔR values measured from marine mammal material and those derived from other local organisms, including marine shell (Gordon and Harkness, 1992; Dyke, McNeely and Hooper, 1996; Dumond and Griffin, 2002). ΔR values measured from seal bones have ranged around 300-500 years in the north Atlantic (Olsson, 1980), 800 years in Antarctica (Koch *et al.*, 2019), but in the absence of knowledge about USNM 375734’s feeding grounds and given concerns with the methodology, no attempt was made to choose a ΔR value to correct measurement Tx-1651.

**References**

Dumond DE, Griffin DG. 2002. Measurements of the Marine Reservoir Effect on Radiocarbon Ages in the Eastern Bering Sea. *ARCTIC*, **55**(1), 77–86. (doi: 10.14430/arctic692)

Dyke AS, McNeely RN, Hooper J. 1996. Marine reservoir corrections for bowhead whale radiocarbon age determinations. *Canadian Journal of Earth Sciences*, **33**(12), 1628–1637. (doi: 10.1139/e96-123)

Gordon JE, Harkness DD. 1992. Magnitude and geographic variation of the radiocarbon content in Antarctic marine life: Implications for reservoir corrections in radiocarbon dating. *Quaternary Science Reviews*, **11**(7–8), 697–708. (doi: 10.1016/0277-3791(92)90078-M)

Haas H, Banewicz J. 1980. Radiocarbon Dating of Bone Apatite Using Thermal Release of CO 2. *Radiocarbon*, **22**(2), 537–544. (doi: 10.1017/S0033822200009863)

Haynes V. 1968. Radiocarbon: Analysis of Inorganic Carbon of Fossil Bone and Enamel. *Science*, **161**(3842), 687–688. (doi: 10.1126/science.161.3842.687)

Koch PL, Hall BL, de Bruyn M, Hoelzel AR, Baroni C, Salvatore MC. 2019. Mummified and skeletal southern elephant seals (*Mirounga leonina* ) from the Victoria Land Coast, Ross Sea, Antarctica. *Marine Mammal Science*, **35**(3), 934-956. (doi: 10.1111/mms.12581)

Morlan R, Nelson DE, Brown TA, Vogel JS, Southon JR 1990. Accelerator Mass Spectrometry Dates on Bones from Old Crow Basin, Northern Yukon Territory. *Canadian journal of archaeology*, 14(1990), 75–92. Available at: https://www.jstor.org/stable/41102449)

Olson EA, Broecker WS. 1961. Lamont Natural Radiocarbon Measurements VII. *Radiocarbon*, **3**(3016), 141–175. (doi: 10.1017/S0033822200020919)

Olsson IU. 1980. Content of 14 C in Marine Mammals from Northern Europe . *Radiocarbon*, **22**(3), 662–675. (doi: 10.1017/s0033822200010031)

Polach HA. 1992. Four Decades of Progress in 14C Dating by Liquid Scintillation Counting and Spectrometry. in *Radiocarbon After Four Decades*. New York, NY: Springer New York, 198–213. (doi: 10.1007/978-1-4757-4249-7_14)

Stuiver M, Braziunas TF. 1993. Modeling Atmospheric 14 C Influences and 14 C Ages of Marine Samples to 10,000 BC. *Radiocarbon*, **35**(1), 137–189. (doi: 10.1017/S0033822200013874)

Taylor RE, Bar-Yosef O. 2014. *Radiocarbon Dating : An Archaeological Perspective*. Second Edi. Taylor & Francis Group. Available at: https://ebookcentral.proquest.com/lib/gmul-ebooks/detail.action?docID=1803095.

Valastro JR., S. *et al.* 1988. University of Texas at Austin Radiocarbon Dates XVI. *Radiocarbon*, 30(2), 197–253.

Valastro JR., S., Mott Davis, E. and Varela, A. G. 1975. University of Texas at Austin Radiocarbon Dates X. *Radiocarbon*, 17(1), 52–98.

Valastro JR, S. and Mott Davis, E. 1970. UNIVERSITY OF TEXAS AT AUSTIN RADIOCARBON DATES VIII. *Radiocarbon*, 12(2), 617–639.

Zazzo, A. and Saliège, J. F. 2011. Radiocarbon dating of biological apatites: A review. *Palaeogeography, Palaeoclimatology, Palaeoecology*. Elsevier B.V., 310(1–2), 52–61. (doi: 10.1016/j.palaeo.2010.12.004.
