# Supplementary material for: Extreme dispersal or human-transport? The enigmatic case of an extralimital freshwater occurrence of a Southern elephant seal from Indiana
Source: PeerJ. 2020 Sep 2;8:e9665. doi: 10.7717/peerj.9665 (PMC7474520; doi:10.7717/peerj.9665)
Supplement: Table S1 — Upper canine measurements in cm for modern specimens of Northern and Southern elephant seals and USNM 375734. Data of Mirounga leonina from Koch et al. (2019). Data of Mirounga angustirostris from this study. [file peerj-08-9665-s003.docx]

**Supplementary Table S1.** Upper canine measurements in cm for modern specimens of Northern and Southern elephant seals and USNM 375734. Data of *Mirounga leonina* from Koch et al. (2019). Data of *Mirounga angustirostris* from this study.

| **Specimen number** | **Species** | **Age** | **Sex** | **Mesiodistal** | **Buccolingual** |
| --- | --- | --- | --- | --- | --- |
| **AMNH 48154** | *Mirounga leonina* | Adult | Female | 1.5 | 1.2 |
| **AMNH 48155** | *Mirounga leonina* | Adult | Female | 1.3 | 1.05 |
| **AMNH 48156** | *Mirounga leonina* | Adult | Female | 1.05 | 0.8 |
| **AMNH 48159** | *Mirounga leonina* | Adult | Female | 1.95 | 1.65 |
| **AMNH 48160** | *Mirounga leonina* | Adult | Female | 1.25 | 1.2 |
| **AMNH 48166** | *Mirounga leonina* | Adult | Female | 1.2 | 0.9 |
| **AMNH 77911** | *Mirounga leonina* | Adult | Female | 1.15 | 1 |
| **AMNH 77912** | *Mirounga leonina* | Adult | Female | 0.95 | 0.85 |
| **AMNH 77913** | *Mirounga leonina* | Adult | Female | 1.25 | 1.05 |
| **AMNH 77926** | *Mirounga leonina* | Adult | Female | 1.7 | 1.25 |
| **CAS 6251** | *Mirounga leonina* | Adult | Female | 1.26 | 1.08 |
| **AMNH 18682** | *Mirounga leonina* | Adult/subad | Male | 3.2 | 2.95 |
| **AMNH 31720** | *Mirounga leonina* | Adult/subad | Male | 3.7 | 3 |
| **AMNH 34904** | *Mirounga leonina* | Adult/subad | Male | 3.6 | 3 |
| **AMNH 48151** | *Mirounga leonina* | Adult/subad | Male | 3.4 | 3.05 |
| **AMNH 48152** | *Mirounga leonina* | Adult/subad | Male | 3.95 | 4.1 |
| **AMNH 48153** | *Mirounga leonina* | Adult/subad | Male | 3.35 | 2.75 |
| **AMNH 48161** | *Mirounga leonina* | Adult/subad | Male | 2.1 | 1.9 |
| **AMNH 48162** | *Mirounga leonina* | Adult/subad | Male | 2.3 | 2.1 |
| **AMNH 70240** | *Mirounga leonina* | Adult/subad | Male | 3 | 2.6 |
| **AMNH 77916** | *Mirounga leonina* | Adult/subad | Male | 2.35 | 2.05 |
| **AMNH 77919** | *Mirounga leonina* | Adult/subad | Male | 3 | 2.8 |
| **AMNH 77923** | *Mirounga leonina* | Adult/subad | Male | 2.5 | 2.35 |
| **AMNH 77928** | *Mirounga leonina* | Adult/subad | Male | 3.65 | 3.05 |
| **CAS 6250** | *Mirounga leonina* | Adult/subad | Male | 3.62 | 3.54 |
| **MCZ 1178** | *Mirounga leonina* | Adult/subad | Male | 3.49 | 2.86 |
| **MCZ 1179** | *Mirounga leonina* | Adult/subad | Male | 4.29 | 3.33 |
| **MCZ 35085** | *Mirounga leonina* | Adult/subad | Male | 3.81 | 3.18 |
| **USNM 241199** | *Mirounga leonina* | Adult/subad | Male | 2.7 | 2.25 |
| **USNM 484893** | *Mirounga leonina* | Adult/subad | Male | 2.6 | 2.4 |
| **USNM 21890** | *Mirounga angustirostris* | Adult/subad | Female | 2.4 | 1.75 |
| **USNM 21738** | *Mirounga angustirostris* | Adult/subad | Male | 4.7 | 3.7 |
| **USNM 260867** | *Mirounga angustirostris* | Adult/subad | Male | 3.55 | 2.55 |
| **USNM 265353** | *Mirounga angustirostris* | Adult/subad | Male | 4.4 | 3.65 |
| **USNM 267987** | *Mirounga angustirostris* | Adult/subad | Female | 2.1 | 1.75 |
| **USNM 38234** | *Mirounga angustirostris* | Adult/subad | Female | 2.3 | 1.4 |
| **USNM 375734** | Cast Indiana (this work) | Adult | Unknown | 4.3 | 3.29 |

Abbreviations: AMNH, American Museum of Natural History; CAS, California Academy of Sciences; MCZ, Museum of Comparative Zoology, Harvard; USNM, Departments of Paleobiology and Vertebrate Zoology (Division of Mammals), National Museum of Natural History, Smithsonian Institution.
