# Supplementary material for: Extreme dispersal or human-transport? The enigmatic case of an extralimital freshwater occurrence of a Southern elephant seal from Indiana
Source: PeerJ. 2020 Sep 2;8:e9665. doi: 10.7717/peerj.9665 (PMC7474520; doi:10.7717/peerj.9665)
Supplement: File S1 [file peerj-08-9665-s001.pdf]

Stromberg, M.W
[illegible]

Amfile
AMFILE FILE AND INDEX CO.
5821 T3

D/9

PURDUE UNIVERSITY
SCHOOL OF VETERINARY SCIENCE AND MEDICINE
LAFAYETTE, INDIANA

DEPARTMENT OF VETERINARY ANATOMY

December 17, 1965

E. Leland Webber
Secr. & Director
Chicago Museum of Natural History
Roosevelt and Lakeshore Drive
Chicago 5, Illinois

Dear Dr. Webber:

A building contractor here in town recently uncovered part of a skull which we were asked to identify. The source appears to be from a depth of about 30 feet in an excavation near the Wabash river. The bone itself is a reasonably intact maxilla with a large intact canine tooth. The five cheek teeth are missing but the alveoli indicate them to have been simple in nature and quite small.

Our search so far points toward some member of the Order Pinnepedia with a likely prospect being the elephant seal.

If we were to ship the specimen to you is it possible that some member of your staff might identify it and also give us an opinion as to how old it may be? The appearance seems to be that of something quite recent but the depth at which it was discovered is a bit puzzling.

Sincerely,

M. W. Stromberg

M.W. Stromberg
Professor and Head

MWS/jjf

Mr. Edward C. Mitchell
Arctic Biol. Station
Fisheries Research Board of Canada
P.O. Box 400
St. Anne de Bellevue
Quebec, Canada

23 December 1965

Professor M. W. Stromberg
Purdue University
School of Veterinary Science and Medicine
Dept. of Veterinary Anatomy
Lafayette, Indiana

Dear Professor Stromberg:

Your letter of 23 December has been referred to me for reply. I suggest that you ship the specimen to me in the Department of Geology at the Museum for identification.

Very truly yours,

William D. Turnbull
Associate Curator,
Fossil Mammals

WDT:JF

Mirounga ~~[illegible]~~ angustirostris
Comp. best with our
M. leonina
from Macquarie, Aust.

PURDUE UNIVERSITY
SCHOOL OF VETERINARY SCIENCE AND MEDICINE
LAFAYETTE, INDIANA

DEPARTMENT OF VETERINARY ANATOMY

December 29, 1965

Mr. William D. Turnbull
Associate Curator, Fossil Mammals
Department of Geology
Chicago Natural History Museum
Roosevelt Road and Lake Shore Drive
Chicago, Illinois 60605

Dear Mr. Turnbull:

Many thanks for your letter and offer to identify the remnant of a skull which was unearthed here in town.

We are enclosing the bone and will be waiting to hear from you.

Sincerely,

M.W. Stromberg

M.W. Stromberg
Professor and Head

MWS/jjf

Enclosure

(1)

January 5, 1966

Professor M. W. Stromberg, Head
Department of Veterinary Anatomy
Purdue University
School of Veterinary Science and Medicine
Lafayette, Indiana

Dear Professor Stromberg:

The elephant seal maxillary arrived in good shape, and as far as I can tell by comparing it with materials in our collection it is indeed an elephant seal. It compares best with Mirounga leonina, which is an Antactic species. The comparison with Mirounga angustirostris (Pacific O. of No. Amer.) is pretty good, probably within the range of individual variation, but the infraorbital foramen in your specimen is more oval and less rounded where it opens to the front. I have not been able to compare it with Cistophora, the hooded seal, which ought to be done.

I have shown the specimen to Dr. ~~George~~ Joseph Moore, Curator of our recent mammals division, and he suggests that we send it to Mr. Edward C. Mitchell, who is in the process of doing a doctoral study at the University of California on this group of pinnepeds. Mr. Mitchell is now with the Arctic Biological Station, Fisheries Research Board of Canada, P. O. Box 400, St. Anne de Bellevue, Quebed, Canada. With your permission, I will send it on to him, from whom I think we can expect to get specific identification.

I would like to ask a few questions concerning the locality of this find. You state that the source was from a depth of about 30 feet in an excavation near the Wabash River. Is it possible to be more specific about the locality, the matrix, and any other details about the situation of its burial? As you point out, the appearance of the bone seems to be quite recent. Is there any possibility that the site could have been part of a dump? It is indeed puzzling that a marine mammal of this sort be found at that depth in sediments at Lafayette, Indiana. It is, of course, known that a few whales managed to get into the Great Lakes during the Post-Pleistocene, but I know of no record of the elephant seal's getting in. Offhand, it would seem most logical that somehow or other man is responsible.

It would be possible to get a carbon 14 date, wlthough bone dates are not too satisfactory and it would require sacrificing some of the speciment, so I would think that the identification should be made first.

PURDUE UNIVERSITY
SCHOOL OF VETERINARY SCIENCE

I notice some very water-worn black pebbles in a number of the pores, and one large one occludes the first post-canine alveolis. Could it possibly have come from a gravel bed and, if so, would it be possible to get a sample of this gravel?

Thank you for calling this puzzling specimen to our attention. I will await your word before either returning the specimen to you or forwarding it to Mr. Mitchell.

Sincerely yours,

W. D. Turnbull
Associate Curator
of Fossil Mammals

WDT:egp

PURDUE UNIVERSITY
SCHOOL OF VETERINARY SCIENCE AND MEDICINE
LAFAYETTE, INDIANA

DEPARTMENT OF VETERINARY ANATOMY

January 13, 1966

Dr. W.D. Turnbull
Associate Curator
of Fossil Mammals
Chicago Natural History Museum
Roosevelt Road and Lake Shore Drive
Chicago, Illinois

Dear Dr. Turnbull:

Many thanks for your reply and the information therein. We would be pleased to have you send the specimen to Mr. Mitchell in Quebec so please feel free to do so.

Meanwhile I shall contact the man who discovered it and gain whatever information I can on the exact location and other pertinent factors. I suspect that a friend from the Soils Dept. may well be of help on this so I will plan to see him also.

You may expect to hear from us as soon as we have this information.

Sincerely,

M. W. Stromberg

M.W. Stromberg
Professor and Head

MWS/jjf

FISHERIES RESEARCH BOARD OF CANADA
ARCTIC BIOLOGICAL STATION

SHIPPING ADVICE

Shipped from

Ste. Anne de Bellevue, P.Q.

TO Dr. William D. Turnbull
Field Museum of Natural History
Roosevelt Road of Lakeshore Drive
Chicago Illinois 60605, USA

We have today shipped to the above address the undermentioned goods

per

AIR FREIGHT .... ~~Collect~~ Charges-Prepaid

(Name of Carrier)

1 Carton containing scientific specimen (1 Jawbone) NO COMMERCIAL VALUE

weight 2 lbs - Dimensions 6" x 6" x 10½"

Date 17 Jan 67

H. W. McNeill
(Signature)

This form to be completed in triplicate, and when shipment is made from the field, mail original to addressee, one copy to Arctic Biological Station, P.O. Box 400, Ste. Anne de Bellevue, Quebec, and retain one for reference.

cc: Thomas Meadows Canada Limited

Stromberg, M.W.

Meadows AIRFREIGHT CANADA LIMITED

IN BOND ADVICE NOTE FOR CUSTOMS

TORONTO, ONT.	MONTREAL, P.Q.	VANCOUVER, B.C.	WINNIPEG, MAN.
AIR CARGO TERMINAL BLDG. RM. 106 · P.O. BOX 25 TORONTO INTERNATIONAL AIRPORT PHONE · 676-2590 TELEX · 02-29583	AIR CARGO BUILDING RM. 117 · P.O. BOX 126 MONTREAL INTERNATIONAL AIRPORT PHONE · 636-3945 TELEX · 01-2748	VANCOUVER INTERNATIONAL AIRPORT PHONE · 278-5158 TELEX · 04-50167	ADMINISTRATION BLDG. ROOM 2578 WINNIPEG INTERNATIONAL AIRPORT PHONE · 772-5958

MESSRS.

Fisheries Research Board of Canada F
Arctic Biological Station
P.O.Box 400
Ste. Anne de Bellevue, Que.

PLEASE QUOTE OUR REFERENCE: EX 587420

DATE Jan. 18/67

THESE CHARGES ARE PAYABLE TO OUR OFFICE AT Montreal

ROUTED	AIRLINE AIRWAYBILL	MANIFEST	GOODS LOCATED AT
BOAC	BOAC YUL 4616077	BA 567/19	

FROM	HOUSE AIRWAYBILL	PART LOT OF
Montreal, Que.	YUL 8948698	

MARKS & NUMBERS / DESCRIPTION / WEIGHT

1 carton Scientific Specimen, 2#, No comm. value
Measurements: 6x6x11, 1#VOL.

STATEMENT OF CHARGES

ADVANCES	U$7.50	$8.11
AIRFREIGHT		
CARTAGE		
ATTENTION		
EXPORT ENTRY		
CUSTOMS INVOICE		
APPRAISAL FEE		

CUSTOMS BROKER:

SHIPPER ☐
CONSIGNEE ☒

Dr. William D. Turnbull
Field Museum of Natural History
Roosevelt Road of Lakeshore Drive
Chicago, Ill., 60605, USA

STORAGE CHARGES ADDITIONAL FOR ACCOUNT OF CONSIGNEE IF NOT CLEARED CUSTOMS AND DELIVERED WITHIN THE FREE TIME.

CERTIFICATION THAT TRANSPORTATION CHARGES ARE COLLECT ☐ OR PREPAID ☒ $ | $8.11

NOTE: WE COVER INSURANCE AS YOUR AGENT ON WRITTEN INSTRUCTIONS ONLY. OUR LIABILITY FOR LOSS AND/OR DAMAGE DOES NEVER EXCEED THAT OF ANY CARRIER WHOSE SERVICES WE MAY USE.

ALL BUSINESS HANDLED IS SUBJECT TO THE CONDITIONS PRINTED ON OUR SHIPPERS DECLARATION FORMS. E.&O.E.

ROUTE VIA MEADOWS AIRFREIGHT

Date....17 Jan 67........................

H. W. McNeill
....
(Signature)

This form to be completed in triplicate, and when shipment is made from the field, mail original to addressee, one copy to Arctic Biological Station, P.O. Box 400, Ste. Anne de Bellevue, Quebec, and retain one for reference.

cc: Thomas Meadows Canada Limited

Stromberg, M.W.

FISHERIES RESEARCH BOARD OF CANADA
ARCTIC BIOLOGICAL STATION

SHIPPING ADVICE

Shipped from

Ste. Anne de Bellevue, P.Q.

TO Dr. William D. Turnbull
Field Museum of Natural History
Roosevelt Road of Lakeshore Drive
Chicago Illinois 60605, USA

We have today shipped to the above address the undermentioned goods per

....AIR FREIGHT.. ~~Collect~~
Charges-Prepaid
(Name of Carrier)

1 Carton containing scientific specimen (1 Jawbone) NO COMMERCIAL VALUE
weight 2 lbs - Dimensions 6" x 6" x $10\frac{1}{2}$"

Date....17 Jan 67........................

H. W. McNeill
....
(Signature)

This form to be completed in triplicate, and when shipment is made from the field, mail original to addressee, one copy to Arctic Biological Station, P.O. Box 400, Ste. Anne de Bellevue, Quebec, and retain one for reference.

cc: Thomas Meadows Canada Limited

7 February 1966

Mr. Edward C. Mitchell
Arctic Biological Station
Fisheries Research Board of Canada
P. O. Box 400
St. Anne de Bellevue, Quebec, Canada

Dear Mr. Mitchell:

I have recently had sent in to me for identification, the maxillary of an elephant seal. The specimen came from about 30 ft. down in sediments along the Wabash River at Lafayette, Indiana. The bone is not re-placed, and therefore looks to be Recent and not fossil, but the location and depth of the find is puzzling. Also there are some black pebbles and sand occluding one alveolus and some small nutrient foramina. I had planned to have our minerologist do some more with these associated materials after get-ting further word from the people at Purdue University about the locality and its geologic setting, but that word has not come in yet.

Meanwhile at Dr. Joseph Moores' suggestion that you ought to see this specimen which I cannot identify to species from our comparative Mirounga materials, I am writing to see if you would care to have me send it on to you.

Sincerely yours,

William D. Turnbull
Associate Curator,
Fossil Mammals

WDT:JF

FISHERIES RESEARCH BOARD
OF CANADA
HEAD OFFICE IN OTTAWA
(UNDER THE MINISTER OF FISHERIES)

OFFICE DES RECHERCHES SUR
LES PÊCHERIES DU CANADA
BUREAU CENTRAL À OTTAWA
(SOUS L'AUTORITÉ DU MINISTRE DES PÊCHERIES)

FILE No. / DOSSIER N° 12-1-2

Arctic Biological Station,
P.O. Box 400,
Ste. Anne de Bellevue, Quebec,
February 15, 1966.

Dear Dr. Turnbull:

Thank you very much for your letter of 7 February and your offer to send along the elephant seal maxillary. Yes, I would be very interested in studying it, for just now I have been concerned with the fossil record of this pinniped, and have some material in hand with which to compare it.

Of course, the locality of your specimen is most unlikely, and would lead me to inquire about elephant seals in circuses and zoos in the area. Do you think that the stratigraphic context can be satisfactorily established?

Sincerely,

Edward Mitchell

Edward Mitchell,
Marine Mammalogist.

Dr. William D. Turnbull,
Associate Curator, Fossil Mammals,
Chicago Natural History Museum,
Roosevelt Road and Lakeshore Dr.,
Chicago, Illinois 60605, U.S.A.

Stromberg, M.W.

[illegible] RESEARCH BOARD

OFFICE DES RECHERCHES SUR
[illegible]ES PÊCHERIES DU CANADA
[illegible]REAU CENTRAL À OTTAWA
[illegible]'AUTORITÉ DU MINISTRE DES PÊCHERIES)

July 5, 1966

18 Feb., 1966

Mr. Edward Mitchell
Marine Mammalogist
Arctic Biological Station
P.O. Box 400
Ste. Anne de Bellevue, Quebec
Canada

Dear Mr. Mitchell:

The specimen is on its way. Your thoughts about the locality being unlikely are exactly mine. I have asked Dr. Stromberg to do what he could to get more information on the stratigraphy, and I intend to have our mineralogist look again at the sand and pebble inclusions in the alveolae, and nutrient foramena. Still no further word on this. Please be careful not to loose any of these sand grains, as they might be a help in this matter.

Sincerely,

William D. Turnbull
Associate Curator, Fossil Mammals

[illegible]onded sooner
[illegible]bruary. Yes,
[illegible]e specifically
[illegible]nish and American)
[illegible]ern species in
[illegible]ons not directly
[illegible]urements from
[illegible]ating original and
[illegible]t you know the

[illegible]e end of the
[illegible] from the Caribbean
[illegible]hins. I hope to
[illegible]anuscript discussing
[illegible]f October.

[illegible]f allowing me to
[illegible]e.

FISHERIES RESEARCH BOARD OF CANADA
HEAD OFFICE IN OTTAWA
(UNDER THE MINISTER OF FISHERIES)

OFFICE DES RECHERCHES SUR LES PÊCHERIES DU CANADA
BUREAU CENTRAL À OTTAWA
(SOUS L'AUTORITÉ DU MINISTRE DES PÊCHERIES)

FILE No. / DOSSIER No 12-1-2

Arctic Biological Station
P.O. Box 400,
Ste. Anne de Bellevue, P.Q.

July 5, 1966

Dr. William D. Turnbull,
Associate Curator, Fossil Mammals,
Chicago Natural History Museum,
Roosevelt Road and Lake Shore Drive,
Chicago, Illinois 60605

Dear Dr. Turnbull:

Please accept my abject apologies for not having responded sooner about the supposed elephant seal snout received in February. Yes, it does look like Mirounga, but I doubt that it can be specifically identified. The literature I've checked (mainly Spanish and American) discusses differences between the northern and southern species in terms of sutural junctions and some cranial proportions not directly related to the snout. Perhaps with a series of measurements from both species it can be allocated. I've been accumulating original and published measurements for this purpose, and will let you know the result as soon as possible.

Might I ask for the loan of the snout until about the end of the year, for I leave in a week for a three month cruise from the Caribbean to Greenland tagging and collecting whales and dolphins. I hope to get back on to the elephant seal specimen (and my manuscript discussing elephant seal historical biogeography) by the end of October.

Thank you for your forebearance, and the courtesy of allowing me to look at the snout. Please give my best to Dr. Moore.

Sincerely,

Ed Mitchell

Edward Mitchell

11 July 1966

Dr. Edward Mitchell
Fisheries Research Board
Arctic Biological Station
P. O. Box 400
Ste. Anne de Bellevue, P.Q.,
Canada

Dear Dr. Mitchell:-

Thank you for your letter of July 5 relating to the Mirounga snout. As far as I am concerned you are welcome to keep the specimen as long as is necessary for its study. However, since it is not mine I have written to Prof. Stromberg at Lafayette, Indiana so that should he or the discoverer need the specimen back earlier than the end of the year, you can be contacted directly.

Enclosed is a copy of an earlier letter to Dr. Stromberg. It gives the tentative conclusions I was able to arrive at from comparisons with our Recent materials in case this be of any help to you.

Have a good field season.

Sincerely,

William D. Turnbull
Associate Curator,
Fossil Mammals

WDT:JF

11 July 1966

Professor M. W . Stromberg
Department of Veterinary Anatomy
Purdue University
School of Veterinary Science and Medicine
Lafayette, Indiana

Dear Professor Stromberg:-

On Friday I received the enclosed letter from Dr. Mitchell and today I replied telling him to keep the _Mirounga_ specimen for the rest of the year providing it was alright with you and its discoverer (copy of letter also enclosed). I took this liberty because of the short interval before his departure for the field. If for any reason you have to have the specimen back sooner write to him immediately.

Did you ever make any headway on the locality, entombing matix etc.?

Sincerely,

William D. Turnbull
Associate Curator,
Fossil Mammals

WDT:JF

AIR MAIL

FISHERIES RESEARCH BOARD OF CANADA
HEAD OFFICE IN OTTAWA
(UNDER THE MINISTER OF FISHERIES)

OFFICE DES RECHERCHES SUR LES PÊCHERIES DU CANADA
BUREAU CENTRAL À OTTAWA
(SOUS L'AUTORITÉ DU MINISTRE DES PÊCHERIES)

Arctic Biological Station,
P. O. Box 400,
Ste. Anne de Bellevue, P.Q.

FILE No.
DOSSIER N° 12-1-2

January 16th, 1967.

Dr. William D. Turnbull,
Associate Curator, Fossil Mammals,
Field Museum of Natural History,
Roosevelt Road at Lake Shore Drive,
Chicago, Illinois 60605,
U.S.A.

Dear Dr. Turnbull:

I have not been able to arrive at a satisfactory identification of the maxillary bone you sent along some months ago. The specimen definitely represents some species of Mirounga, but which one is the question. I have only a small female skull (LACM M1227) of M. angustirostris and a large male skull (BM(NH) 19545.2030) of M. leonina available to me here for comparisons. But reference to a photograph of M. angustirostris (Elliot, 1904, Field Mus. Zool. Ser. 4, 2, pl. LXV) and a drawing of the same species (Allen, 1880, U.S. Geol. Geogr. Surv. Terr., Misc. Publ. 12, fig. 58-Hall and Kelson's fig. 522 is a poor copy of this poor drawing) show that the posterolateral corner of the dorsal surface of the premaxillary projects farther caudally than does the posteromedial corner. The opposite case obtains in the M. leonina skull in my hands. Perhaps this is what Davidson (1929, Proc. Calif. Acad. Sci., 18, 9, p. 236) meant when stating that in M. angustirostris "in the dorsal aspect the premaxillae as they extend backward also expand laterally, the lateral outline being distinctly convex in its basal half. The southern specimens examined all appear to have the lateral margins of the premaxillae parallel."

The specimen definitely needs to be compared with a series of M. leonina skulls. Perhaps Judith King at the British Museum (Natural History) would be willing to do this. I am not now willing to identify the specimen without such a comparison, but feel that it might well prove to be M. leonina. It is being returned to you under separate cover.

Sincerely,

Ed Mitchell

Edward Mitchell.

UNIVERSITY OF CALIFORNIA
Department of Zoology
~~Storer Hall~~
DAVIS, CALIFORNIA 95616

Dr. W. D. Turnbull
Department of Mammals
Field Museum of Natural History
Chicago, Illinois 60600

DAVIS OCT-4'71 CALIF
U.S. POSTAGE 08

H BOARD OF CANADA
JR LES PÊCHERIES DU CANADA
ation, St. Mary's Road,
Bellevue, Que.

12-12

30 September 1971

Paleontology,
istory,
hore Drive,

er the ideas growing out of your
cord in the Mississippi Valley
point where I think it could
ation. I'm just on the fence,
your present thoughts are. I
clined, we could put a short
would be relatively noncommittal.
at the record should be documented.

Sincerely yours,

Ed

Edward Mitchell.

FRB

FISHERIES RESEARCH BOARD OF CANADA
OFFICE DES RECHERCHES SUR LES PÊCHERIES DU CANADA

CANADA

Arctic Biological Station, St. Mary's Road,
Ste. Anne de Bellevue, Que.

12-12

30 September 1971

Dr. William Turnbull,
Department of Vertebrate Paleontology,
Field Museum of Natural History,
Roosevelt Road and Lake Shore Drive,
Chicago, Illinois 60605,
U. S. A.

Dear Bill:

I've been mulling over the ideas growing out of your possible elephant seal record in the Mississippi Valley drainage, and am near the point where I think it could stand the light of publication. I'm just on the fence, as I say, and wonder what your present thoughts are. I believe if you were so inclined, we could put a short manuscript together that would be relatively noncommittal. I'm beginning to think that the record should be documented.

Sincerely yours,

Ed

Edward Mitchell.

FRB

November 16, 1971

Dr. M. W. Stromberg, Professor and Head
Department of Veterinary Anatomy
School of Veterinary Science and Medicine
Purdue University
Lafayette, Indiana 47907

Dear Dr. Stromberg:

I write at this time with regard to our correspondence in 1956-6-7? relating to the maxillary bone of an elephant seal which you sent in to me for confirmation of identity. At this point Dr. Edward Mitchell and I have decided that it is probably a mistake not to investigate the matter further. Accordingly, with your permission, I'd like to get a C-14 date and to ask you again to see if you can give me the details of its occurrence and discovery. To get bone for the C-14 date, I propose to drill into the specimen and to take internal bone for the most part, if this is permissible.

The information that I have is contained in several of your letters, that of December 17, 1965 to our Director (E.L. Webber) where you give a 30' depth from an excavation along the Wabash River. In my January 5, 1966 reply, I asked for more information - exact locality, matrix and other details. Also I asked if a sample of the entombing sediment was taken? You will recall that in your reply on January 13, 1966, you offered to gain whatever information you could for me by contacting the discoverer and someone in your Soils Department whom you thought might be helpful.

I seem to recall too that we had a telephone conversation or two, but I can't find any record or notes relating to these calls and perhaps I've misplaced some detail. At any event if you've any other information please fill me in.

Sincerely,

William D. Turnbull, Associate Curator
of Fossil Mammals
Department of Geology

WDT/wr

In about 1972-3 we got a C-14 date – but it took the entire specimen to do the dating so only the cast remains. Date TX 1561 "1651" of 1260 ± 90 Possibly [illegible]?

and the [illegible]

Stromberg, M.W
and the Purdue Univ.
Mirounga specimen
